# Acute rupture of a sinus of Valsalva aneurysm with dehiscence of the tricuspid valve

**DOI:** 10.1016/j.xjtc.2025.03.014

**Published:** 2025-03-28

**Authors:** Maxwell Marlowe, Jeffrey Weiner, English Flack, Craig Mathis, Karla G. Christian, Garrett N. Coyan

**Affiliations:** a Division of Pediatric Cardiology, Vanderbilt University Medical Center, Nashville, Tenn; b Division of Pediatric Cardiac Surgery, Vanderbilt University Medical Center, Nashville, Tenn; c Department of Biomedical Engineering, Vanderbilt University, Nashville, Tenn

**Figure:**
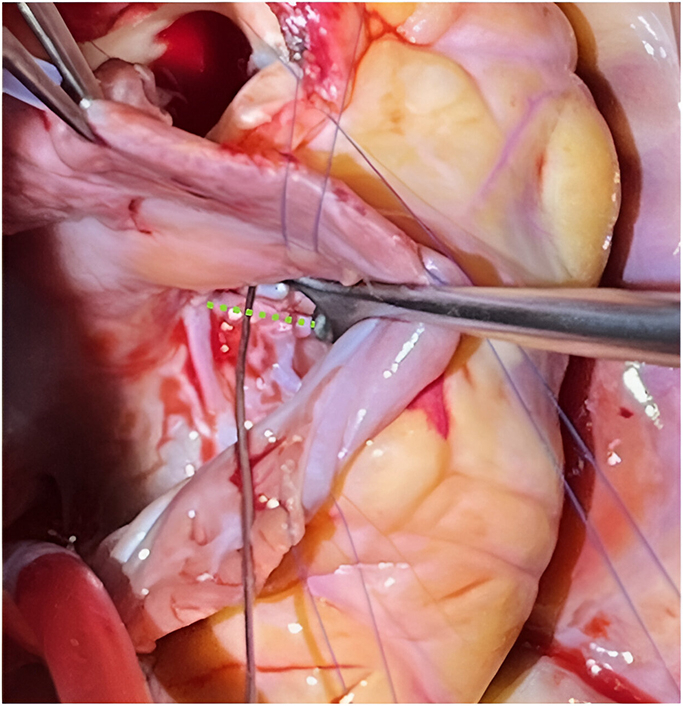
Ruptured SOVA with dehiscence of the tricuspid valve (*dotted green line*).

**Central Message**

A unique case of tricuspid valve dehiscence due to ruptured sinus of Valsalva aneurysm required valve resuspension and commissuroplasty for successful repair.

Ruptured sinus of Valsalva aneurysm (SOVA) can present urgently and most commonly rupture into the right ventricle (RV). We present a unique case of spontaneous rupture of a right SOVA into the atrioventricular junction, leading to dehiscence of the anterior leaflet of the tricuspid valve (TV). Institutional review board approval was not required; informed written consent for publication was obtained.

## Case Report

**Figure 1:**
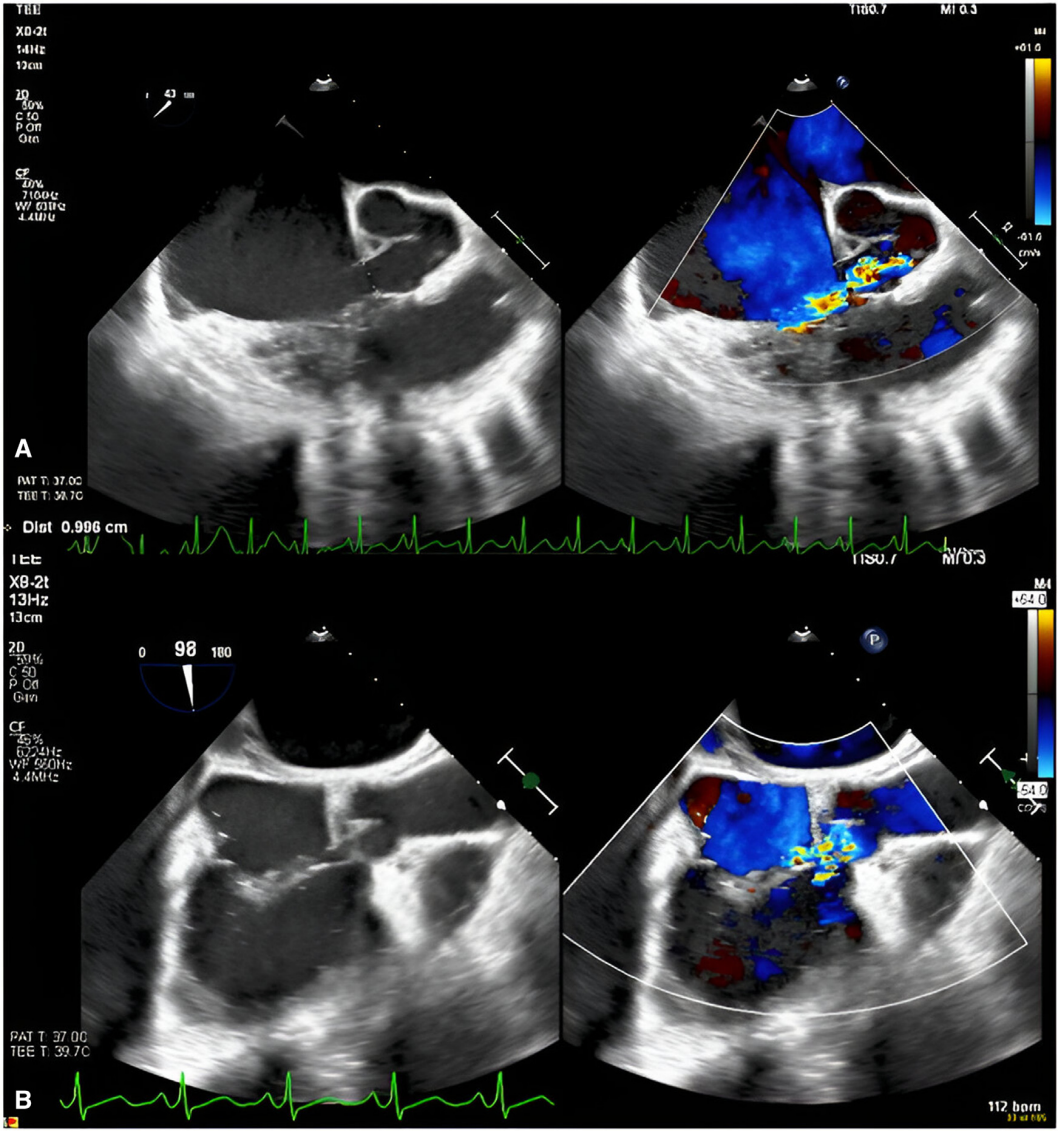
A, TEE depicting an ∼1-cm ruptured SOVA with color flow Doppler demonstrating aliased flow along the apical portion of the tricuspid valve. B, TEE image depicting color flow originating from the ruptured SOVA splaying over the tricuspid valve annulus. The ventricular portion was initially considered a VSD; however, there was no VSD on direct examination, so this was attributable to the flow pattern around the dehiscence defect of the tricuspid valve. *TEE*, Transesophageal echocardiogram; *SOVA*, sinus of Valsalva aneurysm; *VSD*, ventricular septal defect.

An otherwise-healthy 15-year-old male athlete with a previously normal findings on transthoracic echocardiogram (TTE) performed for murmur evaluation and no evidence of infection presented to his primary physician with 3 days of chest pain, tachypnea, orthopnea, and new murmur. TTE obtained upon transfer to our facility demonstrated 9-mm rupture of a right SOVA into the right atrium (RA) with a 7-mm perimembranous ventricular septal defect (VSD). Preoperative transesophageal echocardiogram showed a 1-cm right SOVA ruptured into the RA, mild tricuspid regurgitation (TR), and a perimembranous VSD (Figure 1).

**Figure 2:**
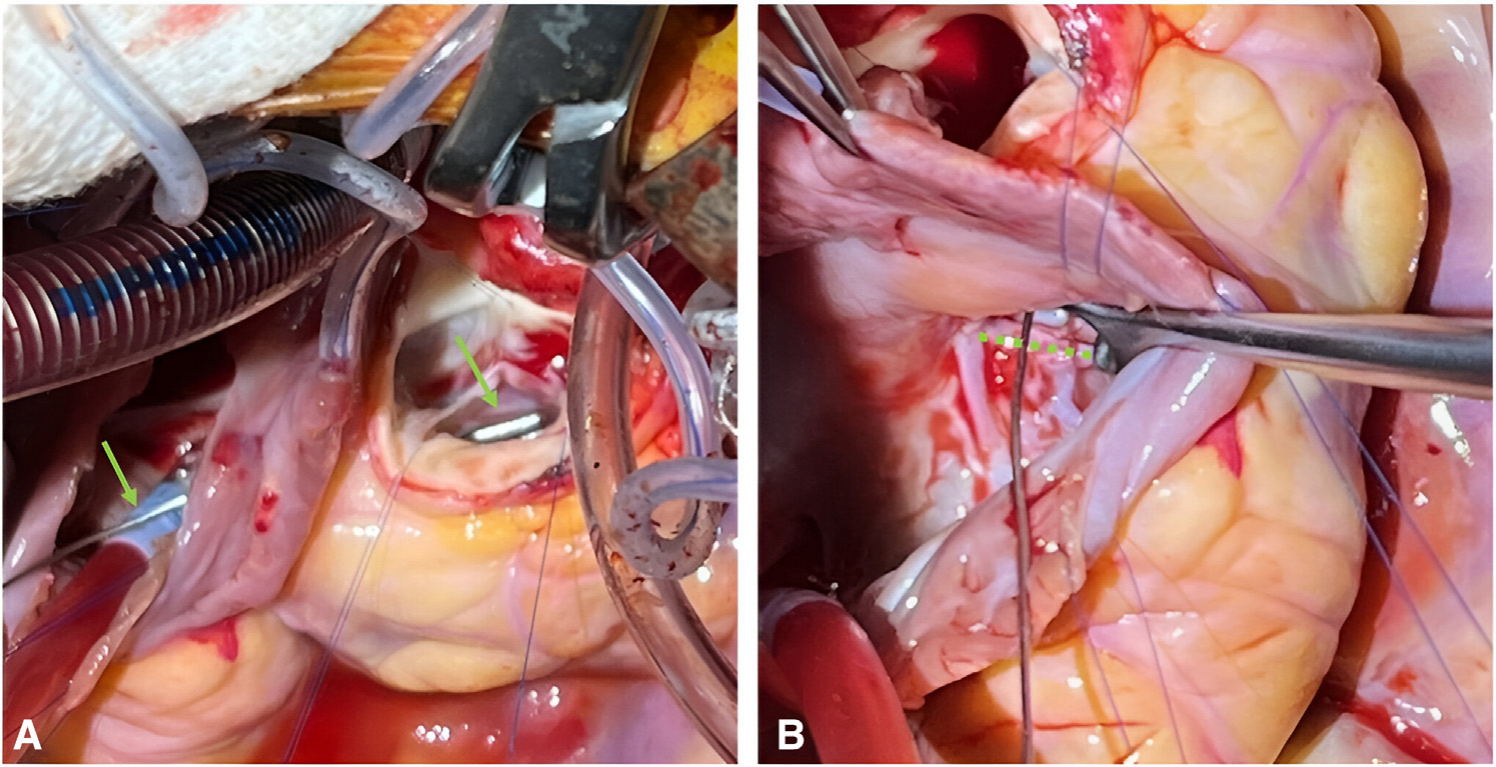
A, Metal coronary probe (*green arrows*) passing from the right atrioventricular junction through the ruptured sinus of Valsalva aneurysm into the right coronary sinus of the aorta after aneurysm sac resection. B, Metal probe passing through the ruptured sinus of Valsalva aneurysm, the anterior septal commissure of the tricuspid valve is detached/delaminated from the annulus (*dotted green line*) in this region.

Intraoperative inspection via the aortic root and RA demonstrated a 1-cm ruptured right SOVA into the RA/RV junction with dehiscence of the TV at the anterior-septal commissure (Figure 2). There was no VSD, and tricuspid chordal apparatus was intact. The internal aneurysmal sac was resected, and the sinus was patched internally from the aortic side with an autologous pericardial patch and a running 6-0 polypropylene suture. The TV was resuspended with two 5-0 polypropylene pericardial-pledgeted mattress sutures passed inferior to superior through the annulus and valve leaflets, sandwiching the SOVA aneurysm tract edges to reenforce closure from the atrial side. Anterior-septal commissuroplasty was performed to improve TV function. The patent foramen ovale was closed before RA closure and weaning from cardiopulmonary bypass.

Postoperative transesophageal echocardiogram showed no residual shunting, trivial to mild TR, and normal biventricular function. The patient was extubated in the operating room, transferred from the intensive care unit on postoperative day 1, and discharged on postoperative day 3.

He was readmitted 2 weeks later with atrial flutter requiring cardioversion and sotalol with subsequent rhythm control. He has returned to baseline functional status, and follow-up TTE demonstrated stable repair and normalized right-sided chamber sizes.

## Discussion

SOVA is rare and can be either congenital or acquired. The most common location of SOVA is the right coronary sinus, most commonly rupturing into the RV. Tricuspid involvement is infrequent and has been rarely described in literature. The most common reports of involvement being associated with the “windsock” of the aneurysm, causing coaptation defect in the valve or high-velocity ruptured SOVA flow, leading to forced systolic opening of the TV.^1^ The effects of SOVA can lead to either TR or stenosis.^2^ One case described the septal leaflet as being “traumatized” but intact, and it was repaired by tricuspid annuloplasty.^3^

This case was unique in that the ruptured SOVA led to dehiscence of the anterior leaflet of the TV requiring surgical resuspension of the TV. The large shunt from the ruptured SOVA and the location near the anterior septal commissure likely led to the suspicion that the shunt was a VSD, but direct inspection revealed this flow pattern was attributable to flow around the dehisced TV. This diagnostic possibility should be considered in acute rupture scenarios and can be addressed by resuspension of the TV while supporting the SOVA repair from the right atrial-ventricular junction. Additional support and repair techniques such as commissuroplasty can also be applied to minimize tricuspid valve regurgitation, and annuloplasty may be indicated to support repairs in patients with a dilated annulus/complex repair. As the result of extreme right-sided chamber dilation, postoperative arrhythmias can also be seen and should be managed per institutional protocols.

## Conflict of Interest Statement

The authors reported no conflicts of interest.

The *Journal* policy requires editors and reviewers to disclose conflicts of interest and to decline handling or reviewing manuscripts for which they may have a conflict of interest. The editors and reviewers of this article have no conflicts of interest.
